# Need for life cycle assessment of pharmaceuticals for kidney healthcare

**DOI:** 10.1007/s10157-025-02647-2

**Published:** 2025-03-19

**Authors:** Kei Nagai, Keisuke Nansai

**Affiliations:** 1 University of Tsukuba Hospital Hitachi Social Cooperation Education Research Center, Hitachi General Hospital, 2-1-1 Jonan-Cho, Hitachi, Ibaraki 317-0077 Japan; 2 Material Cycles Division, National Institute for Environmental Studies, 16-2 Onogawa, Tsukuba, Ibaraki 305-8506 Japan; 3 Department of Nephrology, Faculty of Medicine, University of Tsukuba, 1-1-1 Ten-Nodai, Tsukuba, Ibaraki 305-8575 Japan

**Keywords:** Green nephrology, Carbon footprint, Pharmaceuticals, Life cycle assessment, Sodium-glucose co-transporter-2 inhibitors

## Abstract

**Purpose:**

Global warming is a known risk factor for chronic kidney disease (CKD), and both progression of the disease and its treatment place a burden on the environment. Life cycle assessment (LCA) is an established method for evaluating the global impact of manufactured products, from materials’ procurement to disposal. We aimed to examine available reports of its application to pharmaceuticals.

**Methods:**

A narrative review focused on LCA studies of any pharmaceuticals according to disease area.

**Results:**

We identified the drug types used for treatment of 13 disease areas described in 51 previous LCA studies, classified using the MIDAS database. Among the drug types, anesthetics, inhalants, and antibiotics have received the most attention. However, LCA studies are lacking for the wide range of pharmaceuticals used in kidney healthcare, in the fields of dialysis therapy, treatment of end-stage kidney disease, and associated cardiovascular, metabolic, and endocrine diseases.

**Discussion:**

As the proportion of the population affected by CKD increases, there is a particular urgency for LCA research into drugs administered for their kidney protective effects, such as renin-–angiotensin system inhibitors and sodium-glucose cotransporter 2 inhibitors. As sustainable practices in drug production and the ability to identify and choose effective drugs with low environmental impact require comprehensive LCA data, clinical physicians and pharmacists involved in kidney healthcare should collaborate with pharmaceutical companies to develop an LCA research system .

Incorporating rating of environmental burden of each drug into daily practice is desirable for achieving sustainable kidney healthcare and reducing its environmental impacts.

## Introduction: why should we pay attention to LCA of pharmaceuticals?

Global warming and the deterioration of air and water quality in recent years have led to various health problems [1, 2]. Global warming is a known risk factor for chronic kidney disease (CKD) [3], and the progression of CKD and maintenance dialysis therapy place a heavy burden on the environment [4–6]. Life cycle assessment (LCA) were established for the purpose of analyzing the environmental impact of the entire manufacturing process, from raw material procurement and manufacturing to sales and disposal [7]. All types of global impact can be quantified by LCA, including global warming, air pollution, health hazards, and economic impact. Among the various types of impact, greenhouse gas emissions are the most important and most commonly assessed item (as carbon footprint, CFP) as they are directly linked to climate change. LCA research is well established in some industries, as an effective means for formulating measures and proposals to improve sustainability, but is only just beginning in the field of medicine. Medical professionals involved in kidney healthcare who are concerned about the sustainability of dialysis therapy have been promoting Green Nephrology [1], while it has yet to become widespread in Japan [8]. Several studies focusing on Green Nephrology have reported the results of CFP research on dialysis therapy in various countries [9–16]. Approximately one-third to one-half of the CFP in treatment of dialysis patients is estimated to be derived from pharmaceuticals [11–13]. However, it has also been noted that due to the lack of background CFP information, pharmaceuticals have been deliberately excluded from the “system boundary” that should be calculated as an area of interest in LCA studies [9, 10, 16].

When choosing among drugs with the same clinical efficacy, it is logical from the perspective of Sustainable Development Goals (SDGs) to select those with low environmental impact. Differences in environmental impact are possible when comparing oral drugs with injectable drugs: injectable drugs require high levels of sterilization, as well as refrigeration, metal needles, and other equipment, and have been considered to have a higher CFP than oral drugs. However, there are only modest numbers of case studies of LCA in such pharmaceutical manufacturing process. Among the possible reasons for this situation, one is the issue of confidentiality related to pharmaceutical products and technology, which makes it difficult to obtain CFP inventory information from the companies.

To develop sustainable kidney healthcare, it is a necessary but highly challenging task to calculate the environmental impact of pharmaceuticals as the basis for determining clinical practice patterns for physicians. To address this issue, we investigated the current state of LCA research on drugs and noticed the lack of reports from the perspective of “disease areas of pharmaceuticals”, which is important to clinical physicians. It is possible that the researchers reviewing and summarizing LCA studies have been mainly environmental engineers [17, 18], and have focused on technical issues in the pharmaceutical process and differences in LCA methods (such as the definition of system boundaries) rather than the clinical applications of drugs. In this review, we investigate the problem of the lack of LCA information for pharmaceuticals that play a central role in kidney healthcare, based on a search of published LCA studies.

## Overview of previous LCA studies and mismatches with the pharmaceutical market

A review of articles summarizing previous LCA studies on pharmaceuticals [17–19] identified 51 studies, excluding duplicates. The results of these were examined in detail, along with four studies [20–23] that the authors identified through a manual search. CFP was the main environmental impact indicator used in the studies, some of which also included impact assessments of air pollution, toxicity to humans, acidification of soil, land use, and water consumption [19]. A total of 85 drugs were mentioned. After grouping together drugs that appeared in multiple reports (ibuprofen, 5 reports; acetaminophen, 3 reports; morphine, 2 reports), there were LCA studies on 59 different drugs. Among them, there were seven categories of non-specific substances: enzymes [24], intermediates [25, 26], acids [27], solvents [28–30], active pharmaceutical ingredients (APIs) [31–38], monoclonal antibodies [39–41], and tablets [42], to give a final total of 52 types of drugs.

**Fig. 1:**
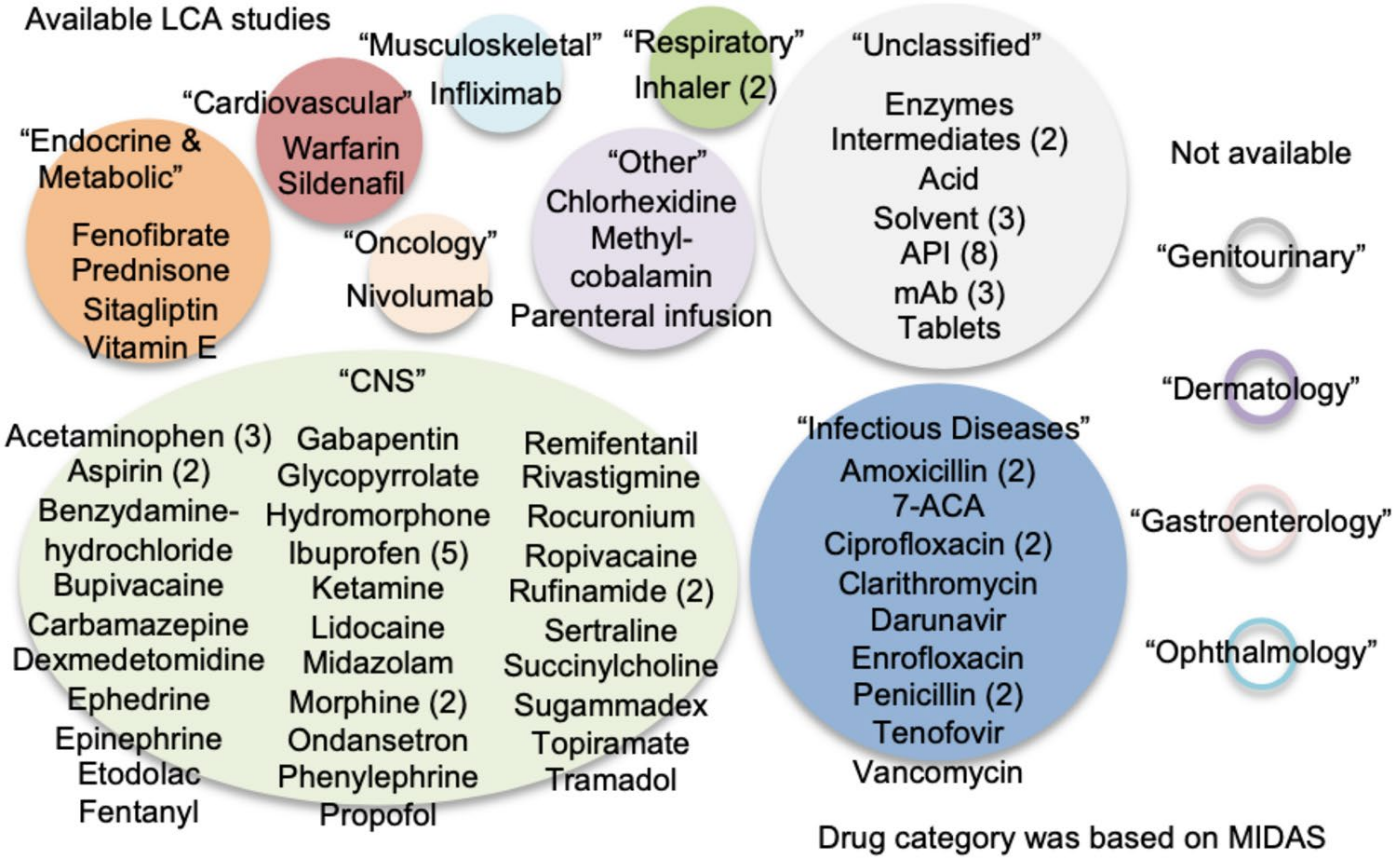
Pharmaceuticals studied in published LCA research, classified according to disease area. The number of studies is shown in parentheses. The disease classification was based on IQVIA MIDAS® [43]

For the purposes of this paper, we classified the disease areas corresponding to each drug based on MIDAS, which is an IQVIA proprietary information service which integrates IQVIA’s national audits into a globally consistent view of the pharmaceutical market, and provides estimated product volumes of registered medicines, trends, and market share through retail and non-retail channels [43]. MIDAS includes 13 major areas (CNS, Infectious diseases, Endocrine & Metabolic, Cardiovascular, Respiratory, Musculoskeletal, Oncology, Genitourinary, Gastroenterology, Dermatology, Ophthalmology, Unclassified, Other) and 600 disease groups. CKD and glomerular disease are categorized as Genitourinary. After classifying the 59 types of drugs that were the subject of LCA research according to disease area (Fig. 1), we identified three areas that have been attracting particular attention since 2012: anesthetics, inhalants, and antibiotics. The motivations for focusing on these areas are as follows.

### Anesthetics

Gases used in anesthesia and intensive care such as sevoflurane, desflurane, isoflurane, and nitrous oxide have a stronger greenhouse effect than CO_2_. Accordingly, there are concerns about their environmental impact [44]. LCA research has led to efforts to reduce the contribution of anesthetic gases to CFP, for example by replacement with intravenous anesthetics such as propofol, which has an environmental impact four orders of magnitude lower than that of nitrous oxide. The need to consider CFP from gaseous anesthesia has led to promotion of LCA research. In our review, the overwhelming majority of reports (15 reports, 31 types) included drugs in the “CNS” category such as anesthetics and painkillers [21, 22, 45–57].

### Inhalers

As they allow the drug to be administered directly to the lungs, inhalers are a necessary treatment for patients with chronic obstructive pulmonary disease and bronchial asthma. However, there are concerns about the greenhouse gases produced by the gas components mixed with the drug. Specifically, pressurized metered-dose inhalers (pMDIs) have a considerably larger CFP than dry powder inhalers (DPIs) [58, 59]. LCA studies investigating the switch from pMDIs to other types of inhalers are currently underway.

### Antibiotics

Antibacterial and antiviral drugs are used all over the world, in agriculture as well as in medicine [60, 61]. Given the increase in demand associated with future population growth, the environmental impact of their production is naturally a concern, and numerous LCA studies have investigated this topic [20, 62–68]. Antibiotics are particularly problematic in terms of their impact on ecosystems and human health due to contamination of water sources and soil [69–71]. Therefore, LCA studies have focused on the environmental impact of antibiotics at the end of life, namely, the processes of disposal and excretion [72, 73].

A very small number of LCA studies were conducted in other disease areas: 4 in Endocrine & Metabolic, 2 in Cardiovascular, 1 in Musculoskeletal, and 1 in Oncology; and none in Genitourinary, Dermatology, Gastroenterology, or Ophthalmology (Fig. 1). According to MIDAS, annual sales in Japan (calculated over August–August) of all pharmaceuticals increased from 10,650 billion yen (BY) in 2019 to 11,689 BY in 2024, which is an increase by 9.7% over 5 years, and the equivalent of about 10% of the entire national budget of 114,381 BY in 2024 (Table 1). When considering LCA from a macro-perspective, the basic approach for pharmaceuticals in current use is to calculate the product of the price of various pharmaceuticals and the basic unit (greenhouse gas emissions per monetary amount) [74]. In other words, the greater the drug sales, the more significant the related environmental impact. According to the 2024 data, sales for CNS (which includes anesthetics) amounted to 918 BY (−10.4% change over 5 years), Respiratory (which includes inhalants) to 769 BY (−1.4%), and Infectious diseases (which includes antibiotics) to 875 BY (+31.1%), making up just 21.9% of the total for the three areas over the 5 years. We cannot ignore the CFP of pharmaceuticals in disease areas that account for the remaining ~80% of the market. To reduce the environmental impact of future medical care, CFP must first be calculated, particularly in areas such as Oncology (2279 BY in 2024, 5-year change rate +43.1%), which is a high-ranking disease area in terms of sales but for which LCA research has not progressed, as well as Cardiovascular (1,242 BY in 2024, −3.2%) and Endocrine & Metabolic (1,340 BY in 2024, +4.3%).

**Table 1:** Trends in annual sales of pharmaceuticals by disease area in Japan

Disease region	2019	2024	2024–2019 (%)
Central nervous system	1024	918	−10.4%
Respiratory	779	769	−1.4%
Infectious diseases	667	875	31.1%
Oncology	1590	2276	43.1%
Endocrine & metabolic	1285	1340	4.3%
Cardiovascular	1283	1242	−3.2%
Musculoskeletal	1115	1014	−9.1%
Genitourinary	479	449	−6.3%
Gastroenterology	697	796	14.2%
Dermatology	339	437	28.9%
Ophthalmology	329	321	−2.6%
Unclassified	859	1039	21.0%
Other	204	213	4.5%
Total	10,650	11,689	9.7%

Unit, billion yen. Author analysis based on IQVIA MIDAS® quarterly value sales data for period MAT Q1 2019 to MAT Q1 2024 reflecting estimates of real-world activity. Copyright IQVIA. All rights reserved. Reprinted with permission

## Unmet needs between kidney healthcare and LCA research

Dialysis therapy is the mode of kidney healthcare that has been attracting the most attention, because of its perceived environmental impact due to consumption of plastic materials such as dialysis membranes and dialysis circuits, as well as water and electricity [5, 75]. However, actual calculations have shown that the environmental impact of dialysis treatment is extremely high, with a CFP related to pharmaceuticals, at around one-third to one-half of the entire CFP of a patient with dialysis program [11–13]. A wide variety of drugs is administered to patients with CKD as the disease progresses, and the CFP calculated based on medical expenses also increases with progression [6, 76]. Despite this situation, there has been no progression of LCA for pharmaceuticals in the field of kidney disease. There is a very wide range of disease areas related to kidney healthcare, and therefore, a variety of pharmaceuticals are used to treat the symptoms; e.g., end-stage kidney disease (diuretics, anemia treatments, potassium binders, and phosphate binders), cardiovascular diseases as complications of CKD (antihypertensive drugs, among others), and metabolic and endocrine diseases (oral hypoglycemic agents and insulin, among others). In addition, the drugs used for primary and secondary glomerular disease are similar to those used for immunological diseases and hematological disorders, and drugs from the oncology field such as rituximab and cyclophosphamide are also often used. Taken together, nephrologists prescribe drugs from a wide range of fields, and it can be said that they cover all disease areas on a daily basis (Table 1). Among these, drugs that are often administered for their kidney protective effects include RAS (renin–angiotensin system) inhibitors [77], and SGLT2 (sodium-glucose cotransporter 2) inhibitors [78, 79]. As the proportion of the population affected by CKD continues to increase, we consider that LCA research into these drugs in particular is an urgent matter.

Examples of alternative measures to reduce CFP include changing from gas anesthesia to intravenous general anesthesia, and changing the inhalation device used for inhalers. Among the many types of RAS inhibitors, angiotensin-converting enzyme inhibitors and angiotensin receptor blockers are used in large quantities globally as kidney protective drugs. For drugs that have the same clinical effects, clinical physicians can choose a drug in the same class that has a smaller CFP. To obtain a basis for this judgment, it is sufficient to perform LCA by comparing the CFPs of at least two drugs. In addition, SGLT2 inhibitors differ from existing kidney protective drugs in that they are used in conjunction with RAS inhibitors [78, 79]. Therefore, it is necessary to prove the absolute advantage of SGLT2 inhibitors; i.e., that the environmental cost of administering the drug is commensurate with the reduced environmental impact from slowing the progression of CKD. A recent study that evaluated the results of a randomized controlled trial confirmed the cost-effectiveness of dapagliflozin from a medical economic perspective [80], indicating that it would probably have the effect of reducing environmental impact. Most recently (Dec. 2024), cooperative research by medical societies and pharmaceutical companies has shown the potential for SGLT2 inhibitors to reduce CFP by improving cardiovascular and renal outcomes [81]. In this study, the details of the LCA that forms the basis for calculating the CFP of SGLT2 inhibitors cannot be directly known, but the figure of 0.035 kg (35 g) CO_*2*_e per tablet released by the pharmaceutical company is used in the calculations [81]. Starting with this research, there may be reports estimating the clinical effects of any other types of SGLT2 inhibitors from each company as a reduction in environmental impact.

It is also necessary to perform LCA for erythropoiesis stimulating agent (ESA) and hypoxia inducible factor-prolyl hydroxylase (HIF-PH) inhibitors, as these are commonly used to treat anemia in end-stage kidney disease. Importantly, CFPs differ greatly depending on the pharmaceutical method used; e.g., as compounds, extracts, or recombinant proteins produced by biological methods [19]. Synthetic compounds are created using chemical catalysts from non-renewable carbon sources such as fossil fuels, and extracts are mainly purified molecules extracted from medicinal plants and other natural sources. Semi-synthetic compounds are created by chemically modifying precursors produced by microorganisms or extracted from plants, whereas biological methods use genetic modification and cultivation techniques to extract proteins and ingredients from plants, microorganisms and cells. The CFP of synthetic compounds has been reported as 351 kgCO_2_eq/kg and that of semi-synthetic compounds or extracts as 710 kgCO_2_eq/kg; in contrast, that of pharmaceuticals produced using biological methods is reported to be 29,900 kgCO_2_eq/kg [19]. In terms of reducing environmental impact, the chemical compound daprodustat (C_19_H_16_N_2_O_5_) is expected to be superior to darbepoetin alfa (C_800_H_1300_N_228_O_244_S_5_), a recombinant protein obtained through biological methods, but at present, there is no LCA research to support a conclusion. It is also necessary from the perspective of SDGs to consider whether drugs with a low environmental impact should be actively selected for treatment with ESA or HIF-PH inhibitors, after giving full consideration to clinical indications and contraindications.

## Proposal for achieving decarbonization in pharmaceuticals

The Japanese government has set a target of achieving carbon neutrality by 2050. All 12 of Japan’s leading pharmaceutical companies are planning to reduce their CFP by 20–55% between 2025 and 2030, and have demonstrated sufficient motivation and results to contribute to decarbonization of the industry [82]. If LCA is promoted with the aim of reducing the environmental impact of pharmaceuticals and realizing a decarbonized society, the government should apply preferential measures for pharmaceuticals for which LCA has been conducted. In this context, the environmental assessment method itself must be transparent. Only when LCA is developed in accordance with global standards will it be possible to compare scenarios and carry out environmentally friendly healthcare. For example, as mentioned above, the CFP of biological products is overwhelmingly large due to the method of drug manufacture, but the most important items in the breakdown are the method of cell culture and the energy used in the production process [19]. In contrast, for extraction and chemical synthesis of products that have a small CFP, the CFP of the subsequent sterilization and packaging processes is greater than that of the drug purification process itself [51]. If a drug has a short shelf life and a high disposal rate, or requires metal needles, the disposal cost will be high, and it will be necessary to calculate the cost up to the end of life. Therefore, the consistency of “system boundary”, which defines the scope of the life cycle to which the assessment is applied, is important. One of the reviews evaluated in the present article focused on the system boundary, but of the 37 papers included, there was inconsistent coverage of the same scopes of interest, from synthesis of the active pharmaceutical ingredient (Cradle to API), to packaging and shipping of the drug (Cradle to gate), to its use and disposal after sale (Cradle to grave) [18]. As well as having transparency and consistency, the environmental assessment must be feasible. If we attempted to analyze the entire pharmaceutical manufacturing process as an area of interest, according to the CFP published by representative Japanese pharmaceutical companies, approximately 80% of the CFP is due to greenhouse gases emitted by such as distribution channels and affiliated companies (referred to as Scope 3), and accurately investigating each of these processes requires a great deal of effort [82]. In addition, if the CFP for factory operations is recorded as the CFP for a drug, it is quite rare for a factory to manufacture only a single type of drug, and so the CFP would be calculated for multiple types of drugs, which would be a cause of inaccuracy. Therefore, in the survey of pharmaceutical companies, it is necessary to establish a standard LCA that is performed identically by each company, after setting an identical system boundary that reconciles the ideal with reality.

As well as their value regarding policy recommendations, the results of LCA for pharmaceuticals can potentially have a direct effect on physicians’ clinical practice patterns. Accordingly, these results and appropriate information should be published in academic journals, so they are easily accessible to physicians. Designing pharmaceuticals with a low environmental impact will force pharmaceutical companies to bear economic burdens that are not essential to the manufacturing process, such as procuring raw materials that have low environmental impact, investing in factory facilities that use renewable energy, and considering waste disposal methods. In addition, it is important to avoid a situation in which responsible companies that pursue environmental impact reduction suffer losses or poor business performance. Taken to the extreme, it might be necessary to have a policy that permits only drugs that have been publicly evaluated using LCA or that have been recognized for their efforts to reduce CFP (regardless of the amount of CFP) to be sold on the market. Otherwise, there will be an increasing number of companies that pursue profit alone, without paying attention to environmental issues, and companies that sincerely consider the sustainability of the environment and health will fall behind.

To response consumers’ environmental consciousness, most of the industrial products on market discloses rating of electricity and water consumptions in recent years. In addition, some people dare to use transportation options, such as buses, trains, and airplanes based on information by common search systems to assume total CFP to reach their destinations. We consider pharmaceutical sector should not be exception in regards such environmental rating practice. In some facilities, the price of medicine is displayed when doctors prescribe it using electronic medical records, and if the CFP is displayed in the same way, it will give more practitioners the opportunity to think about environmental issues. Patients with high environmental consciousness and might proactively choose the one with smaller CFP if there are alternative drugs that have the almost same effects and prices. In case of Japan, prescribed drugs are commonly given to patients in pharmacies with an instruction document to inform proper dosage and possible side effects and any cautions. Additional information of estimated CFP of each drug can help not only improve environmental friendly actions by patients, leading to avoid leftover medication for reducing environmental burden of healthcare.

## Conclusion: toward decarbonized kidney healthcare

As global warming and environmental degradation continue to progress, for the sake of sustainable healthcare it is important that pharmaceutical companies, medical professionals, patients, and governments understand the environmental impacts associated with pharmaceuticals. Medical professionals and pharmaceutical companies already work together to treat diseases and improve the health of patients. In the case of CKD, preventing progression of the disease and avoiding hospitalization will additionally reduce the impact of the disease on the environment. In this context, RAS inhibitors and SGLT2 inhibitors are expected to contribute to balancing human health and the environment, due to their effectiveness in preventing the progression of CKD and the onset of cardiovascular disease. Could medical associations and academic societies recommend undertaking LCA research and summarize the obtained results for use in policy recommendations? If the government were to provide incentives for environmentally friendly pharmaceuticals, wouldn’t it be possible to achieve the decarbonized society that we should be working toward? Although it is difficult to specify the types of pharmaceuticals that should be the focus of LCA research, because these depend on the specialist area of the individual clinical physician, previous LCA research into anesthetics, inhalers, and antibiotics provides a good starting point. At the very least, we recognize the importance of LCA of drugs related to CKD because of the increasing prevalence of CKD in aging populations and because its progression leads to an increase in CFP. For this reason, we consider that more LCA research systems that fill unmet needs should be established as soon as possible, ideally by cooperation between medical societies and pharmaceutical companies as shown in few leading achievements [81, 83]. In the end, we hope that both medical professionals and patients will become more aware of the need to choose drugs that are environmentally friendly to protect kidney health.

## Data Availability

The data that support the findings of this study are available on reasonable request from the corresponding author (K. Nagai).
